# Retrospective Identification of Early Autochthonous Case of Crimean-Congo Hemorrhagic Fever, Spain, 2013

**DOI:** 10.3201/eid2706.204643

**Published:** 2021-06

**Authors:** Ana Negredo, María Sánchez-Ledesma, Francisco Llorente, Mayte Pérez-Olmeda, Moncef Belhassen-García, David González-Calle, María Paz Sánchez-Seco, Miguel Ángel Jiménez-Clavero

**Affiliations:** Instituto de Salud Carlos III, Madrid, Spain (A. Negredo, M.P. Sánchez-Seco, M. Pérez-Olmeda);; Hospital Universitario de Salamanca, Salamanca, Spain (M. Sánchez-Ledesma, M. Belhassen-García, D. González-Calle);; Animal Health Research Center, INIA-CISA, Valdeolmos, Spain (F. Llorente, M.A. Jiménez-Clavero);; Centro de Investigación Biomédica en Red de Epidemiologia y Salud Pública, Madrid (M.A. Jiménez-Clavero)

**Keywords:** Crimean-Congo hemorrhagic fever virus, CCHF, CCHFV, antibodies, emerging communicable diseases, parasites, ticks, vector-borne infections, Spain

## Abstract

Before this report, 7 autochthonous human cases of Crimean-Congo hemorrhagic fever had been reported in Spain, all occurring since 2016. We describe the retrospective identification of an eighth case dating back to 2013. This study highlights that the earliest cases of an emerging disease are often difficult to recognize.

Crimean-Congo hemorrhagic fever (CCHF) is a widely distributed tickborne disease in humans, emerging in different parts of the world (*1*). In Western Europe, the first and currently only country affected by this disease is Spain, where the etiologic agent, Crimean-Congo hemorrhagic fever virus (CCHFV) (family *Nairoviridae*, genus *Orthonairovirus*), was first identified in ticks in 2010 (*2*). Of note, the first autochthonous cases of CCHF were reported in 2016. In this hitherto first incidence, the index case-patient presumably acquired the infection from a tick bite, whereas a nurse (secondary case-patient) became infected while caring for the index patient (*3*). Since then, 5 more CCHF cases have been reported (Table): 2 in 2018 (1 of them retrospectively diagnosed in 2019) and 3 more in 2020 (*4*,*5*). All these cases (except the nosocomial case in 2016) arose in summer in rural areas of west-central Spain; 5 occurred in the southernmost part of the autonomous community of Castile and León. Field studies have confirmed that these areas are at risk for CCHF occurrence because of the abundance of *Hyalomma lusitanicum* tick vectors; CCHFV has been verified in specimens collected there, and high seroprevalences have been observed in wild and domestic animals (*4*).

**Table:** Human cases of Crimean-Congo hemorrhagic fever reported to date, in chronological order, Spain

Year	No. cases	Autonomous community/province	Reference
2013	1	Castile and León/Ávila	This study
2016	2	Castile and León/Ávila (index case); community of Madrid/Madrid (secondary case)	(*3*)
2018	2	Extremadura/Badajoz; Castile and León/Salamanca	(*4*)
2020	3	Castile and León/Salamanca	(*5*)

In August 2020, we were contacted by a person who recovered from a severe disease in May 2013, described as “caused by a tick bite,” that occurred in the high-risk region referenced previously, and the etiology remained unknown. The patient’s occupation did not expose her to animals, and she stated that she had not noticed any tick bites since then. The case was suggestive enough to warrant review of the patient’s medical history: 3 days after being bitten by a tick during a walk through the mountains (40°18′26.8′′N, 5°40′40.7′′W), the patient (then a 32-year-old previously healthy woman) sought medical care after experiencing fever and chills. The patient’s general condition worsened the next day (arthromyalgia, nausea, vomiting, and diarrhea), and she was admitted to a local hospital. Physical examination revealed erythema (Figure, panels A, B) and a necrotic lesion on the patient’s back in the area of the tick bite (Figure, panel C). Platelet count dropped from 136,000/µL to 17,000/µL in 3 days, accompanied by remarkable leukopenia and neutropenia. Her general condition deteriorated rapidly and she experienced anasarca, gum bleeding, petechiae, and melena; she was transferred to a tertiary hospital.

**Figure:**
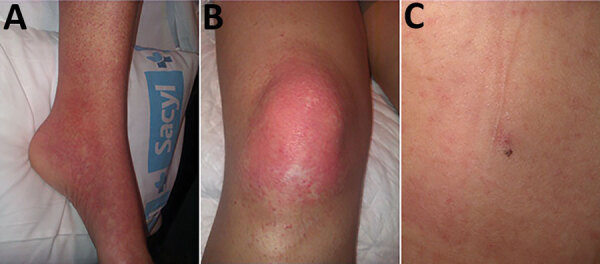
Retrospectively identified early autochthonous case of Crimean-Congo hemorrhagic fever in a woman in Spain, 2013. A, B) Erythema in the patient’s ankle (A) and knee (B) 3 days after a tick bite during a leisure walk. C) Necrotic lesion on patient’s back at site of tick bite.

Laboratory findings included pancytopenia, hypoalbuminemia, and hyperbilirubinemia with elevated transaminases (aspartate aminotransferase [AST] <4,000 U/L [reference range 0–33U/L] and alanine aminotransferase [ALT] <1,000 U/L [reference range 0–32 U/L]). Intracytoplasmic inclusions (morulae) were described in buffy coat examination.

Despite treatment, septic shock occurred, and supportive treatment was started in the intensive care unit. After 10 days of hospitalization, the patient recovered and was discharged.

Final laboratory diagnostic tests ruled out infection by most common tickborne illnesses (i.e., *Rickettsia* spp., *Borrelia burgdorferi*, *Anaplasma* spp., and *Ehrlichia* spp.) and other suspected etiologies (i.e., cytomegalovirus, *Coxiella* spp., hepatitis C virus, hepatitis B virus, HIV). Stool and blood cultures were negative.

At the time of discharge CCHF was not suspected, probably because this disease had never occurred in Spain or other nearby countries, and buffy coat examination suggested ehrlichiosis. Evidence indicates CCHFV was present in 2010 in ticks ≈150 km from the location where the patient was bitten (*2*), but this finding was not deemed medically relevant at that time. However, examined retrospectively, and with the perspective of 7 CCHF cases in 4 years in Spain, 5 of them in the same area, the case strongly suggested CCHFV infection. In agreement with the patient, a new serum sample was collected and tested by the ID Screen CCHF Double Antigen Multi-species ELISA (ID-Vet, https://www.id-vet.com). The serum sample tested positive for antibodies to CCHFV, further confirmed by Crimean-Congo fever virus Mosaic 2 indirect immunofluorescence test for CCHFV-GPC and CCHFV-N, yielding positive results to both GPC and N antigens (EUROIMMUN, https://www.euroimmun.com). Meanwhile, we located and analyzed whole blood and serum samples that were collected 10 days after symptom onset and subsequently stored. CCHFV genome was detected in blood by nested PCR (*3*) and real-time reverse transcription PCR (*6*), whereas CCHFV-N–specific IgG and IgM were found in serum by indirect immunofluorescence test as described previously. Thus, the most likely cause of the disease suffered by the patient in 2013 was CCHF.

This study demonstrates that the occurrence of CCHF cases in Spain started >3 years before the previously reported first known case (Table). This case is the second to be identified retrospectively (*4*), so it would be possible that additional CCHF cases dating even earlier might be diagnosed in the future, since antibodies seem to be long-lasting (>7 years). CCHF should be included in the differential diagnosis after tick bites in areas in which it is endemic. Furthermore, awareness of CCHF is key to prevent nosocomial infections among exposed healthcare workers.
